# A Robust Protocol to Increase NimbleGen SeqCap EZ Multiplexing Capacity to 96 Samples

**DOI:** 10.1371/journal.pone.0123872

**Published:** 2015-04-14

**Authors:** Ilse M. van der Werf, R. Frank Kooy, Geert Vandeweyer

**Affiliations:** Department of Medical Genetics, University of Antwerp, Antwerp, Belgium

## Abstract

Contemporary genetic studies frequently involve sequencing of a targeted gene panel, for instance consisting of a set of genes associated with a specific disease. The NimbleGen SeqCap EZ Choice kit is commonly used for the targeted enrichment of sequencing libraries comprising a target size up to 7 Mb. A major drawback of this commercially available method is the exclusive use of single-indexing, meaning that at most 24 samples can be multiplexed in a single reaction. In case of relatively small target sizes, this will lead to excessive amounts of data per sample. We present an extended version of the NimbleGen SeqCap EZ protocol which allows to robustly multiplex up to 96 samples. We achieved this by incorporating Illumina dual-indexing based custom adapters into the original protocol. To further extend the optimization of cost-efficient sequencing of custom target panels, we studied the effect of higher pre-enrichment pooling factors and show that pre-enrichment pooling of up to 12 samples does not affect the quality of the data. To facilitate evaluation of capture efficiency in custom design panels, we also provide a detailed reporting tool.

## Introduction

Over the last years next-generation sequencing has been applied in many (human) genetics studies. Whole-exome sequencing is an effective technique to screen the great majority of the genes in the genome for the presence of sequence alterations in an unbiased fashion. In medical genetics, it is predominantly used to identify causal genes for genetic disorders, including neurodevelopmental disorders such as intellectual disability and autism spectrum disorders [1–3]. For these purposes the advantages of an unbiased candidate gene discovery outweigh the disadvantages of incomplete coverage of the target region. Indeed, even when sequencing at high depth, a significant number of genes typically remain insufficiently covered in whole-exome sequencing experiments [4]. When the objective is to screen a relatively limited set of known genes with absolute coverage for sequence abnormalities, such as required in routine diagnostics, targeted sequencing of a set of selected genes is more robust.

Among the commercially available technologies, hybridization based in-solution capture (e.g. NimbleGen SeqCap EZ (Roche, Basel, Switzerland) or SureSelect Target Enrichment (Agilent Technologies, Santa Clara, CA, USA)) and amplicon based enrichment (e.g. HaloPlex (Agilent Technologies)) are most widely used. Although all support specific selection of a custom region of interest (ROI), major differences in sequencing efficiency exist. Sequencing efficiency is reflected in the amount of sequencing data needed to reach sufficient coverage of the complete ROI, and is mainly represented by the fraction of enriched DNA fragments belonging to the designed target. For example, the enzymatic restriction-site based HaloPlex design principle typically leads to a significant extension of the captured region, measuring up to twice the original ROI [5]. As a consequence, a major proportion of sequencing capacity is lost to reads flanking the ROI. A second important aspect of enrichment efficiency is the stability of the assay. Despite frequent use of average coverage as a global quality threshold, it does not guarantee an even distribution of coverage depth over the whole target region. When enrichment efficiency differs widely within the assay, high average coverage, and thus absolute sequencing capacity is needed to achieve sufficient coverage of the complete ROI. A metric to compare assay stability is the percentage of bases in the ROI covered by a set fraction of the average base coverage (typically 0.2*$\overset{-}{X}$). Although assay stability can vary between different assays of the same technology, amplicon based enrichment from HaloPlex consistently has a lower stability (e.g. 90% of ROI at 0.04*$\overset{-}{X}$ in [6]) compared to hybridization based capturing (e.g. 97.5% of ROI at 0.1*$\overset{-}{X}$ in [7], 98.7% of ROI at 0.2*$\overset{-}{X}$ in this study). Within the hybridization-based technologies, Nimblegen SeqCap enrichment produces a near-normal distribution of the per-base coverage, whereas Agilent SureSelect coverage distribution shows a heavy tail towards higher values, indicating overrepresentation of a significant proportion of the targets [8]. Finally, further direct comparison of Agilent and NimbleGen in-solution capture assays demonstrated a narrower insert size range and a lower inter-capture variability in coverage for NimbleGen SeqCap EZ, indicating a slightly more robust technique [8]. Based on these studies, the NimbleGen SeqCap EZ technology can be considered to have the highest target enrichment efficiency for both exome and small panel enrichment [4, 8].

There is however a major downside to SeqCap EZ enrichment as the standard workflow only provides labeling of samples with single index adapters, which limits the maximum multiplexing capacity to 24 samples per sequencing experiment. As even the capacity of the low throughput Illumina MiSeq system (Illumina, San Diego, CA, USA) by far exceeds the requirements for multiplexing 24 samples enriched for small target sizes, this leads to excessive coverage and unnecessary experimental costs. Using MiSeq V2 chemistry, one could sequence a target region of up to 1.6 Mb in a single multiplexed 96 sample run with 2x150 bp sequencing to obtain an average coverage of at least 30X, a commonly used cut-off for reliable variant calling [9]. By alleviating the multiplexing limitation, more cost-efficient sequencing can be achieved for the kit showing the highest target enrichment efficiency of the commercially available products.

We present a method to enable 96 sample multiplexing using NimbleGen SeqCap EZ Choice enrichment and dual-indexed sequencing libraries based on ‘home-made’ dual-index adapters and blocking oligos. The extended protocol described here is based on a combination with the KAPA library preparation kit for Illumina platforms (Kapa Biosystems, Wilmington, MA, USA), to enable sequencing on a MiSeq platform. Next to the expansion of the multiplexing capacity, we tested different pre-enrichment pooling factors to reduce the sequencing costs per sample even further. For SeqCap EZ Human Exome library v3.0, pre-enrichment pooling of 4 samples is recommended by the manufacturer, while they demonstrated successful pre-enrichment pooling of 8 samples for panel enrichment [10]. Here, we applied pre-enrichment pooling of 4, 8, 10 and 12 samples and evaluated the impact of the presented protocol on the data quality through detailed coverage analysis reports and genotyping concordance.

## Methods

### Preparation of ‘home-made’ dual-index adapters and blocking oligos

The most recent dual-index adapter sequences were derived from the Illumina sequence letter [11] (Oligonucleotide sequences © 2007–2013 Illumina, Inc. All rights reserved. Derivative works created by Illumina customers are authorized for use with Illumina instruments and products only. All other uses are strictly prohibited) and ordered with HPLC purification. Required modifications are a 5’-phosphate group for D7 adapters and a phosphothiorate bond between the last two nucleotides on the 3’ end for D5 adapters. The lyophilized oligos were dissolved in annealing buffer (10 mM Tris-HCl, pH8.0, 10 mM NaCl, 1 mM EDTA) to a final concentration of 200 μM. By mixing each adapter pair in equal volumes (e.g. 10 μl D5 + 10 μl D7), all 96 possible index combinations were prepared. Formation of the Y-shaped adapter dimers was initiated by heating the mixtures to 95°C in a thermal cycler for 5 minutes, followed by cooling down to 4°C at a rate of 0.1°C/s. Finally, the mixtures were diluted with elution buffer (10 mM Tris-HCl, pH8.0) to a concentration of 10 μM, as specified in the NimbleGen protocol for starting the enrichment protocol with 1 μg of DNA (protocol adapted from [12]). D5 blocking oligos were designed to have the same sequences as the respective D5 adapters, whereas for D7 blocking oligos the reverse complement of the respective D7 adapter sequences was used. All blocking oligos were modified with a 3’ inverted dT and SAGE purified. The lyophilized oligos were dissolved in molecular biology grade water (5Prime, Hilden, Germany) to a final concentration of 100 μM.

**Fig 1:**
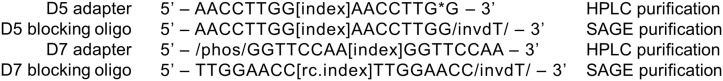
Schematic structural representation of the adapter and blocking oligo sequences and the required modifications. Full-length sequences can be derived from the Illumina sequence letter [11]. *Index = sequence of 6–8 nucleotides that makes each adapter unique*, * = phosphothiorate bond, */invdT/ = inverted dT*, */phos/ = phosphate group*, *rc*. *= reverse complement*. Oligonucleotide sequences © 2007–2013 Illumina, Inc. All rights reserved. Derivative works created by Illumina customers are authorized for use with Illumina instruments and products only. All other uses are strictly prohibited.

A schematic representation of the structure and modifications of the adapter and blocking oligo sequences is provided in Fig 1.

### Proof-of-principle experiment

**Fig 2:**
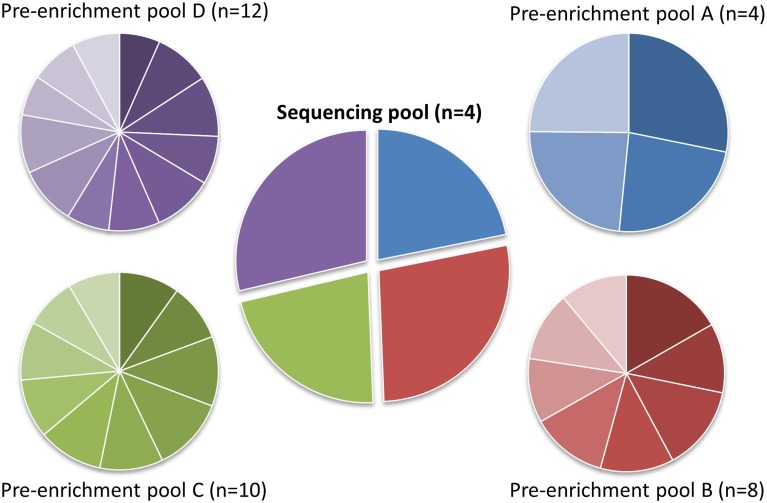
Read distributions by pool, based on the percentage of reads per index. Pre-enrichment pools were pooled before target capture. Sequencing pool consists of the 4 pre-enrichment pools, combined in a single sequencing run.

As a proof-of-principle we multiplexed and sequenced 34 DNA samples that were enriched using a custom NimbleGen SeqCap EZ Choice kit. The DNA samples were obtained from blood, and selected from a follow-up research cohort which was approved by the Ethics committee of the Antwerp University Hospital and the University of Antwerp (EC file 13/1/13). No additional consent was needed, as the data were analyzed anonymously for this study. For each sample, one microgram of high quality genomic DNA was fragmented with a Covaris M220 instrument, using screw-cap microtubes (Covaris, Woburn, MA, USA). Library preparation was performed with the KAPA Library Preparation Kit for Illumina platforms (Kapa Biosystems). The manufacturer’s DNA sample preparation protocol for Roche NimbleGen SeqCap EZ Choice and Exome products (KAPA Biosystems, KR0935—v1.14) was followed, using the ‘home-made’ dual-index adapters at step 6.1 of the protocol. To further reduce experimental costs, we evaluated the impact of pre-enrichment pooling using different numbers of samples. We prepared 4 pre-enrichment pools by equimolarly pooling 4, 8, 10 and 12 samples (Fig 2, pre-enrichment pool A-D respectively). The enrichment panel design, generated using the online NimbleDesign tool (Roche) contained 18 genes with a cumulative target size of 157 kb and a predicted target gene coverage of 99.8%. NimbleGen SeqCap EZ Choice library enrichment was performed according to the manufacturer’s protocol (NimbleGen SeqCap EZ Library SR User’s Guide, version 4.2). At chapter 5 step 4 of the protocol, the appropriate ‘home-made’ blocking oligos were used. After performing the SeqCap EZ enrichment protocol, the four pools were equimolarly pooled (Fig 2, sequencing pool) to a single pool that was sequenced with a 2x150 bp MiSeq run using MiSeq v2 chemistry (Illumina). The full sequences used for the ‘home-made’ adapters and blocking oligos are available upon request; the indices used in this experiment are summarized in S1 Table.

### Data-analysis

Quality control and mapping of the raw sequencing reads was achieved using a slightly modified version of our exome sequencing pipeline [3]. In short, adapter sequences were removed with Cutadapt v.1.2.1 [13] after which bases with a Phred score <30 were removed from the 3’ read end with an in-house developed tool [3]. The remaining high-quality reads were mapped against the reference genome with BWA-MEM v.0.7.4 [14]. PCR-duplicates were removed with Picard v1.88 [15] and the mapping was optimized with GATK v.2.8.1 indel realignment and base quality recalibration [16, 17]. Variants were called with GATK Unified Genotyper after which annotation, filtering and interpretation of the variants was done using VariantDB [18].

### Genotype Concordance

Illumina SNP-array data were previously obtained from 30 samples using a HumanCytoSNP-12 v2.1 beadchip on an iScan system, following standard protocols as provided by the manufacturer (Illumina). SNP genotypes were extracted from Illumina GenomeStudio (v2011.1) using the Genotyping module (v.1.9.4). Concordance between NGS and SNP-array genotypes was defined as identical calls with both techniques.

### Evaluation of Target Capture performance

**Fig 3:**
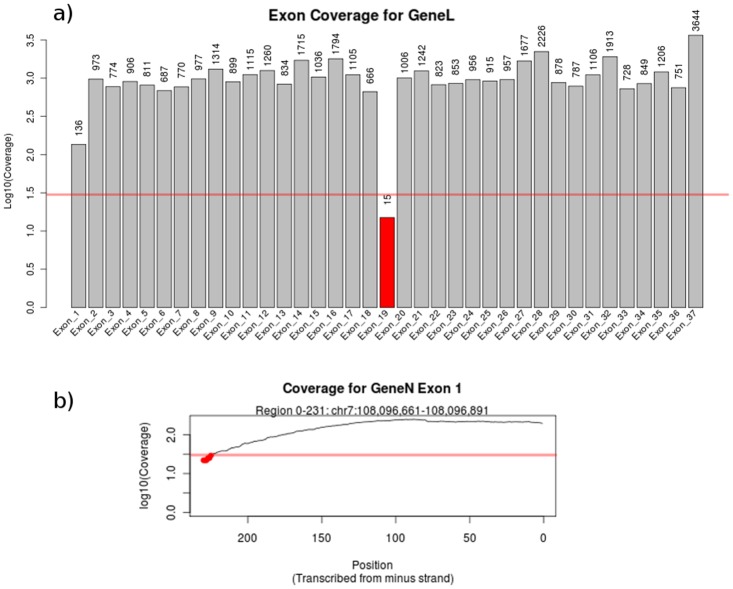
Sections from a coverage report. A) Representation of exon coverage, grouped by gene based on information in the provided BED file. The horizontal red line indicates a user-provided coverage threshold. B) Coverage at base level for one exon, allowing the identification of local drops in sequencing depth.

Target enrichment was evaluated using an in-house tool, available in both a standalone version and through integration with galaxy (https://toolshed.g2.bx.psu.edu/view/geert-vandeweyer/coverage_report) [19–21]. Total read count and mapping performance is calculated using samtools v.0.1.19 [22]. Coverage is calculated within regions of interest using BedTools v.2.17.0 and transformed to on-target mapping performance, average coverage at exon- and base-level, the percentage of bases covered by at least a selected depth and enrichment stability, represented by the percentage of bases covered by at least 0.2 times the average read depth [23]. Results are presented in a PDF report, including the samtools and transformed bedtools values, and per-gene charts of exon coverage (Fig 3a). The user can also request per exon plots of base level coverage, to inspect or identify small drops in coverage depth (Fig 3b). An example of the resulting coverage report is shown in S1 Fig.

## Results

Analysis of the sequencing data revealed that all included indices were recognized and were well distributed over the pools and the respective samples within a pool (Fig 2). The minor variation in distribution falls within the range of standard pipetting inaccuracies. S2 Fig summarizes the coverage distribution per exon over all samples, demonstrating that in our design only four of the 360 targeted exons repeatedly drop below the 30X coverage threshold. A detailed description of the dataset is provided in Table 1. Independent sample Kruskal-Wallis tests indicated significant differences between the means of all groups for each measure. These differences can largely be explained by the different depth of coverage for each pool. As the pools were equimolarly pooled for sequencing, each pool represents approximately one quarter of the sequencing output. Every pool consists of different numbers of samples causing individual samples to be sequenced with different depth, depending on the number of samples in the pool. A lower sequencing depth will by definition result in a lower mean base coverage, a trend that we indeed observe when increasing the number of samples per pool. With a decrease in sequencing depth, one may also expect a decrease in the percentage of bases with more than 30X coverage. Indeed, a small decrease in the percentage of bases with more than 30X coverage was detected when increasing the pooling factor, however the resulting percentage remains very high and is not significantly different between pools C and D (n = 10 or 12 respectively, p = 0.088, post-hoc Fisher’s least significant difference test). The stability of the assay is reflected in the percentage of reads with a coverage that is higher than 20% of the average coverage. This percentage is significantly higher in pool A compared to the other pools (p<0.001), but the means of the three larger pools do not differ significantly (p>0.25, post-hoc Tamhene test), from which we can conclude that higher pre-pooling factors do not severely affect the stability of the assay. The percentage of reads mapped on-target is less dependent on the sequencing depth and mainly depends on the efficiency of the capture reaction. As a result, this parameter can fluctuate between different enrichment designs and between different capture reactions, but should be in the same order of magnitude for the same design. As each pool was used for a separate capture reaction, the average reads mapped on-target per pool slightly differ, but independent of the pooling factor. In fact, the pool with the largest pooling factor (pool D) has the highest mean percentage of reads mapped on-target, with a value of 79.4%. In addition to inter-capture stability, the average percentage of on-target reads is also within range of libraries prepared with the standard NimbleGen SeqCap EZ single-index adapters and enriched using NimbleGen SeqCap EZ Human Exome Library v3.0 in our laboratory (68.4%,±7.8% (S.D.)). These results show that the ‘home-made’ blocking oligos against the dual-index adapter sequences successfully prevented the enrichment of aspecific targets in the custom enrichment, without compromising the enrichment stability over the target region. To test whether pre-enrichment pooling affects the genotyping quality, we compared the sequencing data with SNP array data that were previously obtained for 30 of the 34 samples. A total of 17 SNPs (Table 2) were shared between both datasets, all 17 SNPs in all 30 samples were concordant, suggesting that higher pre-enrichment pooling factors do not influence the genotyping quality.

**Table 1:** Detailed description of the dataset.

	Mean base coverage ± S.D.	Percentage of bases with >30X coverage ± S.D.	Percentage of reads mapped on target ± S.D.	Percentage of bases with coverage > 0.2*average coverage ± S.D.
Pool A (n = 4)	553 ± 51	99.75 ± 0.02	77.2 ± 0.5	99.02 ± 0.04
Pool B (n = 8)	243 ± 37	99.26 ± 0.20	74.4 ± 0.5	98.69 ± 0.10
Pool C (n = 10)	157 ± 18	98.69 ± 0.22	75.2 ± 0.3	98.64 ± 0.12
Pool D (n = 12)	180 ± 26	98.80 ± 0.22	79.4 ± 0.5	98.60 ± 0.08
Total (n = 34)	232 ± 127	98.99 ± 0.41	76.6 ±2.2	98.68 ± 0.16

**Table 2:** Overview of SNPs shared by the targeted enrichment sequencing data and the array data.

Chromosome	Position	rs ID	# of concordant calls between SNP array and NGS data
1	204988535	rs2794866	30/30
2	166020295	rs2304710	30/30
2	166060498	rs920402	30/30
2	166845794	rs7577411	30/30
2	166905375	rs1542484	30/30
7	107789927	rS349077	30/30
7	107824678	rs6970656	30/30
7	107880612	rs1269634	30/30
8	133134877	rs9297840	30/30
8	133139755	rs977939	30/30
10	61831984	rs11599164	30/30
10	61900356	rs6479694	30/30
11	17796992	rs12421233	30/30
11	17803711	rs1236205	30/30
11	118005119	rs868344	30/30
12	52184271	rs303815	30/30
20	62070966	rs2297385	30/30

## Discussion

Many studies illustrated the importance of next-generation sequencing and especially whole-exome sequencing to identify causative mutations in genetic diseases [3, 24, 25]. However, despite the gradual decrease of experimental costs, whole-exome sequencing is still only sporadically used in routine diagnostics as the costs remain relatively high. Targeted screening using next-generation sequencing platforms is a cost-efficient alternative if the desired sequencing capacity to identify the disease-causing mutation is relatively limited in comparison with whole-exome sequencing. This is for instance the case in the diagnostic testing for disorders with a limited genetic heterogeneity. Moreover, the more complete coverage achieved with targeted sequencing results in higher sensitivity and specificity rates, important factors in routine diagnostics. Another major advantage of targeted screening over whole-exome sequencing, especially in diagnostic settings, is the reduced risk of finding unanticipated or incidental findings. These refer to the discovery of mutations in disease genes unrelated to the disorder for which a diagnosis is requested. Major ethical discussions are ongoing within the genetic community about whether or not to report these incidental findings to the patients [26]. By only studying the genes known to be involved in the emergence of the phenotype of a patient, the risk of finding unsought mutations is significantly reduced. Besides the benefits of targeted sequencing in a diagnostic setting, the technique can also be a very interesting alternative for whole-exome sequencing in research projects focusing on specific disorders or pathways as more samples can be sequenced at a lower cost.

In summary, we increased the multiplexing capacity from 24 to 96 samples for NimbleGen SeqCap EZ Choice enrichment, by demonstrating that the dual-indexing of KAPA libraries is compatible with this enrichment protocol using a custom set of adapter and blocking oligos. Furthermore, we showed that pre-enrichment pooling of up to 12 samples is feasible and does not lead to significant data loss per sample, which decreases the costs per sample even further. The total costs per sample are reduced with a factor 2 for a 192 sample project, using this extended protocol compared to the standard NimbleGen SeqCap EZ protocols, and can be reduced even further when including more samples (S3 Fig). Hence, this enhanced protocol provides a robust and high-throughput screening alternative of target regions up to 7Mb, reducing experimental costs, limiting the risk for incidental findings, and increasing sensitivity and specificity rates.

## Supporting Information

S1 Table Overview of the pool distribution and indices. D-codes refer to Illumina Index identifiers as described in the Illumina Sequence Letter, version August 2014. (http://support.illumina.com/downloads/illumina-customer-sequence-letter.html, Oligonucleotide sequences © 2007–2013 Illumina, Inc. All rights reserved.)
(PDF)

S1 Fig Example of a coverage report. The coverage report encompasses the target region coverage and cumulative normalized base-coverage plots, general statistics of the alignment, summary plots of the exon coverage per gene and detailed plots of exons that (partially) failed to reach the applied coverage threshold.
(PDF)

S2 Fig Coverage distribution per gene. For each gene a graph is depicted, showing the mean coverage per exon over all samples (n = 34). Error bars reflect ±1 standard deviation. The commonly used threshold of 30X coverage is indicated in each graph with a black line.
(PDF)

S3 Fig Cost-comparison of NimbleGen single-indexing vs. the presented dual-indexing protocol. (PDF)

S1 Dataset Coverage reports of all samples, containing all data required for the analyses presented here. (GZ)
