# Supplementary material for: A Robust Protocol to Increase NimbleGen SeqCap EZ Multiplexing Capacity to 96 Samples
Source: PLoS One. 2015 Apr 14;10(4):e0123872. doi: 10.1371/journal.pone.0123872 (PMC4397063; doi:10.1371/journal.pone.0123872)
Supplement: S1 Table — D-codes refer to Illumina Index identifiers as described in the Illumina Sequence Letter, version August 2014. (http://support.illumina.com/downloads/illumina-customer-sequence-letter.html, Oligonucleotide sequences © 2007–2013 Illumina, Inc. All rights reserved.) (PDF) [file pone.0123872.s001.pdf]

S1 Table: Overview of the pool distribution and indices. D-codes refer to Illumina Index identifiers as described in the Illumina Sequence Letter, version August 2014. (http://support.illumina.com/downloads/illumina-customer-sequence-letter.html, Oligonucleotide sequences © 2007-2013 Illumina, Inc. All rights reserved.)

Sample	Pool	Index
1	A	D501-D708
2	A	D502-D708
3	A	D503-D708
4	A	D504-D708
5	B	D505-D708
6	B	D506-D708
7	B	D507-D708
8	B	D508-D708
9	D	D501-D709
10	D	D502-D709
11	D	D503-D709
12	D	D504-D709
13	B	D505-D709
14	B	D506-D709
15	B	D507-D709
16	B	D508-D709
17	D	D501-D710
18	D	D502-D710
19	D	D503-D710
20	D	D504-D710
21	C	D505-D710
22	C	D506-D710
23	C	D507-D710
24	C	D508-D710
25	D	D501-D711
26	D	D502-D711
27	D	D503-D711
28	D	D504-D711
29	C	D505-D711
30	C	D506-D711
31	C	D507-D711
32	C	D508-D711
33	C	D505-D712
34	C	D506-D712
