# Supplementary material for: A Robust Protocol to Increase NimbleGen SeqCap EZ Multiplexing Capacity to 96 Samples
Source: PLoS One. 2015 Apr 14;10(4):e0123872. doi: 10.1371/journal.pone.0123872 (PMC4397063; doi:10.1371/journal.pone.0123872)
Supplement: S1 Fig — The coverage report encompasses the target region coverage and cumulative normalized base-coverage plots, general statistics of the alignment, summary plots of the exon coverage per gene and detailed plots of exons that (partially) failed to reach the applied coverage threshold. (PDF) [file pone.0123872.s002.pdf]

Coverage Report for “Sample_5"

Overall Summary

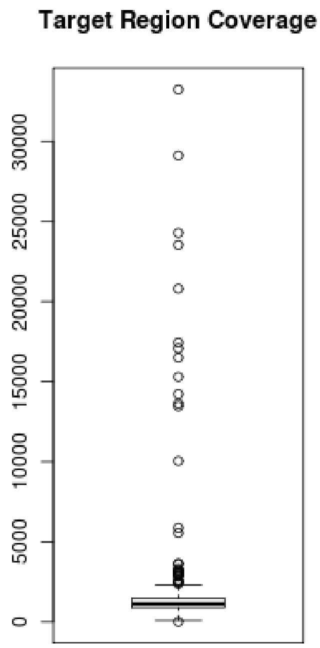

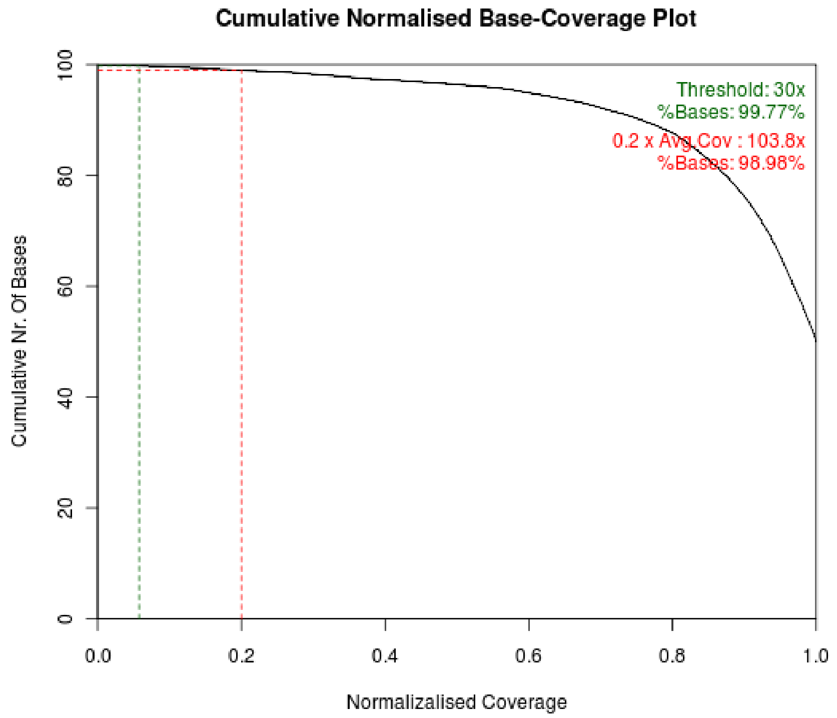

Samtools Flagstat Summary	
In total (QC-passed reads)	934147
Duplicates	0
Mapped (99.93%)	933507
Paired in sequencing	932753
Read1	466245
Read2	466508
Properly paired (98.71%)	920678
With itself and mate mapped	932085
Singletons (0.02%)	190
With mate mapped to a different chr	9568
With mate mapped to a different chr (mapQ≥5)	8018

Target Region Coverage	
Number of Target Regions	360
Minimal Region Coverage	15
25% Region Coverage	904
50% (Median) Region Coverage	1124.5
75% Region Coverage	1489
Maximal Region Coverage	33239
Average Region Coverage	1907
Mapped On Target	73.6
**Target Base Coverage**	
Number of Target Bases	153387
Average Base Coverage	519
Non-Covered Bases	33

Gene Summaries

Legend:

RED: Coverage did not reach set threshold of 30

ORANGE: Coverage was incomplete for the exon. Overruled by red.

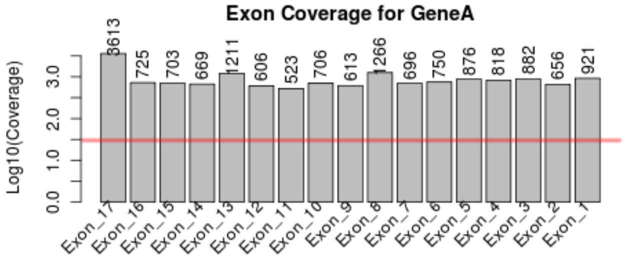

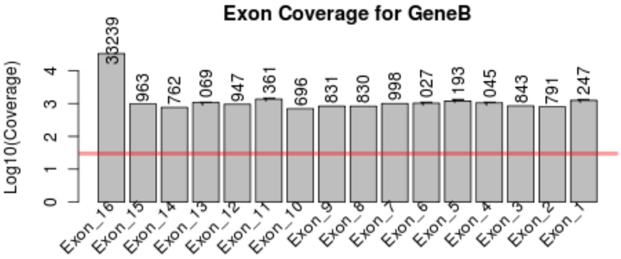

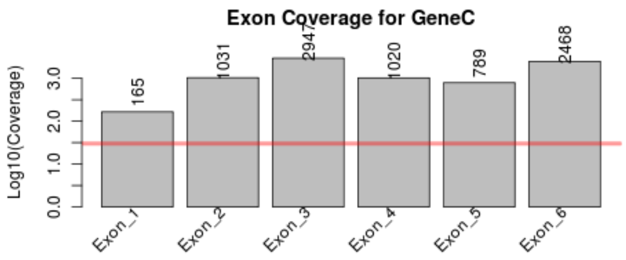
Exon Coverage for GeneC
Log10(Coverage)
0.0
1.0
2.0
3.0
165
1031
2947
1020
789
2468
Exon_1
Exon_2
Exon_3
Exon_4
Exon_5
Exon_6

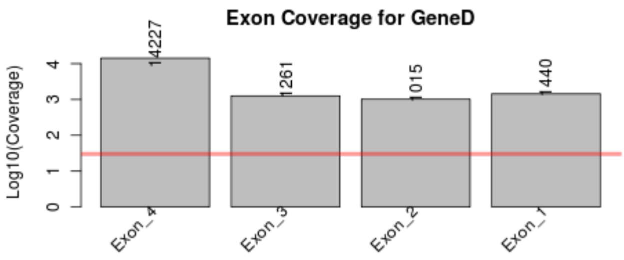
Exon Coverage for GeneD
Log10(Coverage)
0
1
2
3
4
14227
1261
1015
1440
Exon_4
Exon_3
Exon_2
Exon_1

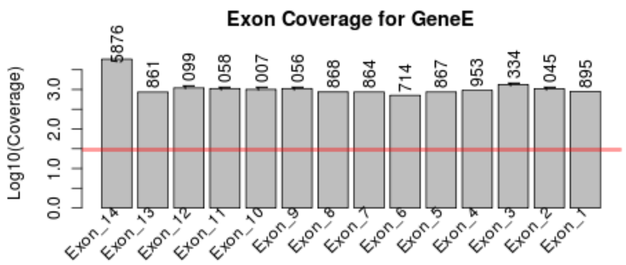
Exon Coverage for GeneE
Log10(Coverage)
0.0
1.0
2.0
3.0
5876
861
1099
1058
1007
1056
868
864
714
867
953
1334
1045
895
Exon_14
Exon_13
Exon_12
Exon_11
Exon_10
Exon_9
Exon_8
Exon_7
Exon_6
Exon_5
Exon_4
Exon_3
Exon_2
Exon_1

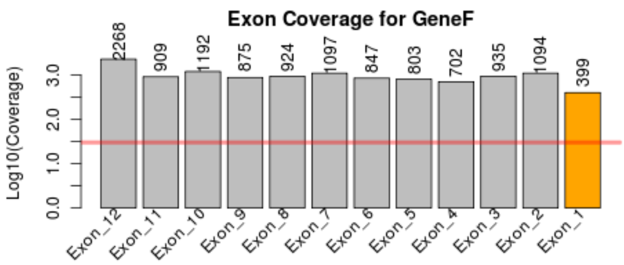
Exon Coverage for GeneF
Log10(Coverage)
0.0
1.0
2.0
3.0
2268
909
1192
875
924
1097
847
803
702
935
1094
399
Exon_12
Exon_11
Exon_10
Exon_9
Exon_8
Exon_7
Exon_6
Exon_5
Exon_4
Exon_3
Exon_2
Exon_1

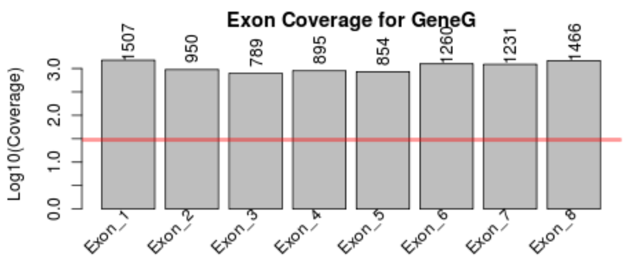
Exon Coverage for GeneG
Log10(Coverage)
0.0
1.0
2.0
3.0
1507
950
789
895
854
1260
1231
1466
Exon_1
Exon_2
Exon_3
Exon_4
Exon_5
Exon_6
Exon_7
Exon_8

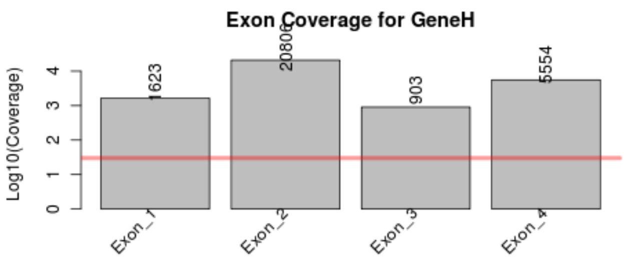
Exon Coverage for GeneH
Log10(Coverage)
0
1
2
3
4
1623
20806
903
5554
Exon_1
Exon_2
Exon_3
Exon_4

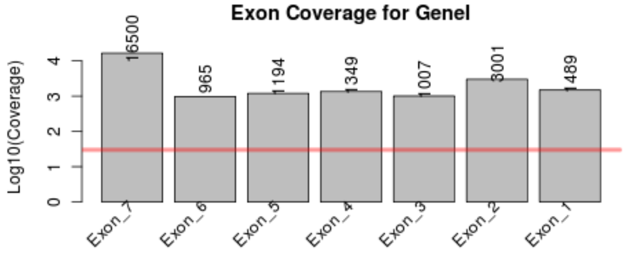
Exon Coverage for GeneI
Log10(Coverage)
0 1 2 3 4
16500 965 1194 1349 1007 3001 1489
Exon_7 Exon_6 Exon_5 Exon_4 Exon_3 Exon_2 Exon_1

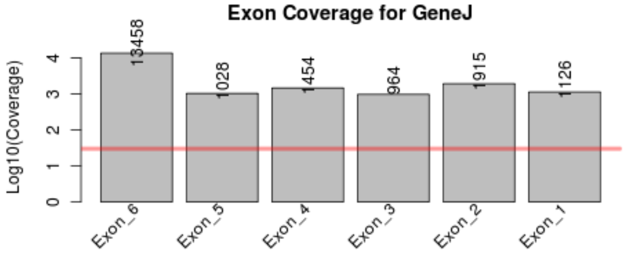
Exon Coverage for GeneJ
Log10(Coverage)
0 1 2 3 4
13458 1028 1454 964 1915 1126
Exon_6 Exon_5 Exon_4 Exon_3 Exon_2 Exon_1

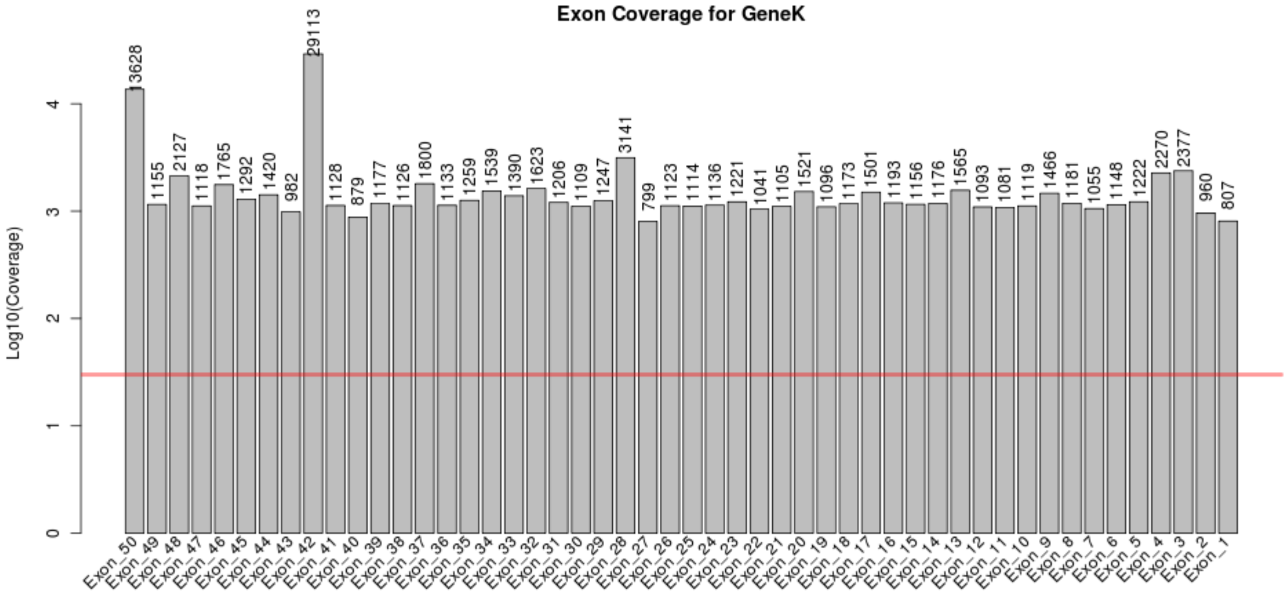
Exon Coverage for GeneK
Log10(Coverage)
0 1 2 3 4
13628 1155 2127 1118 1765 1292 1420 982 29113 1128 879 1177 1126 1800 1133 1259 1539 1390 1623 1206 1109 1247 3141 799 1123 1114 1136 1221 1041 1105 1521 1096 1173 1501 1193 1156 1176 1565 1093 1081 1119 1466 1181 1055 1148 1222 2270 2377 960 807
Exon_50 Exon_49 Exon_48 Exon_47 Exon_46 Exon_45 Exon_44 Exon_43 Exon_42 Exon_41 Exon_40 Exon_39 Exon_38 Exon_37 Exon_36 Exon_35 Exon_34 Exon_33 Exon_32 Exon_31 Exon_30 Exon_29 Exon_28 Exon_27 Exon_26 Exon_25 Exon_24 Exon_23 Exon_22 Exon_21 Exon_20 Exon_19 Exon_18 Exon_17 Exon_16 Exon_15 Exon_14 Exon_13 Exon_12 Exon_11 Exon_10 Exon_9 Exon_8 Exon_7 Exon_6 Exon_5 Exon_4 Exon_3 Exon_2 Exon_1

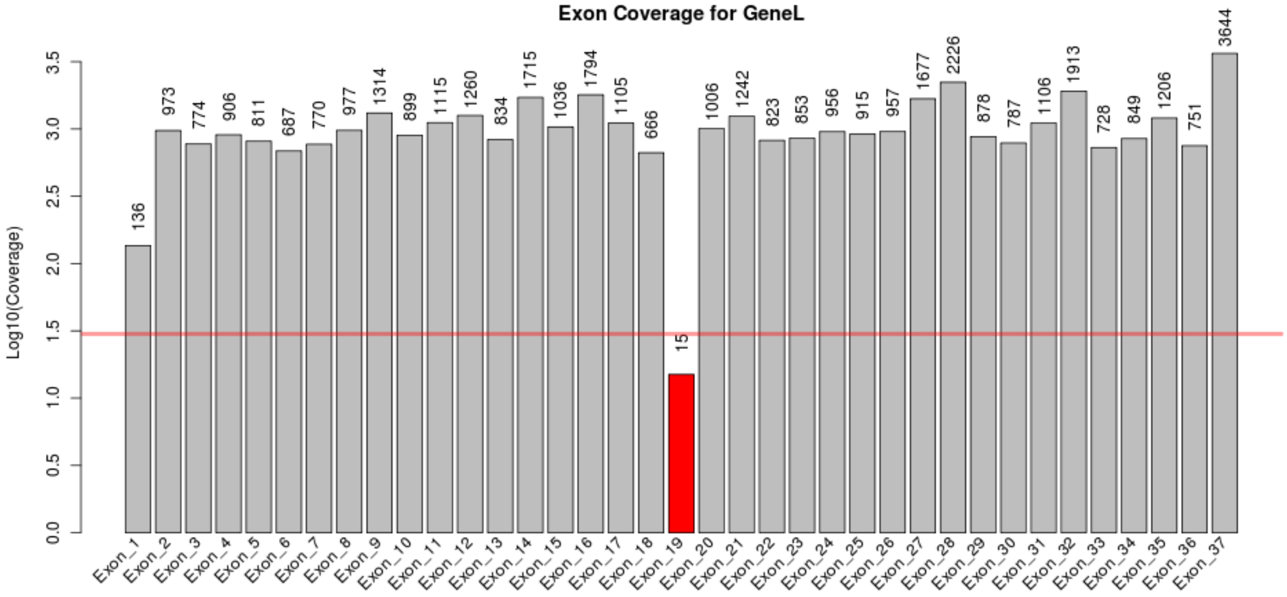
Exon Coverage for GeneL
Log10(Coverage)
0.0 0.5 1.0 1.5 2.0 2.5 3.0 3.5
136 973 774 906 811 687 770 977 1314 899 1115 1260 834 1715 1036 1794 1105 666 15 1006 1242 823 853 956 915 957 1677 2226 878 787 1106 1913 728 849 1206 751 3644
Exon_1 Exon_2 Exon_3 Exon_4 Exon_5 Exon_6 Exon_7 Exon_8 Exon_9 Exon_10 Exon_11 Exon_12 Exon_13 Exon_14 Exon_15 Exon_16 Exon_17 Exon_18 Exon_19 Exon_20 Exon_21 Exon_22 Exon_23 Exon_24 Exon_25 Exon_26 Exon_27 Exon_28 Exon_29 Exon_30 Exon_31 Exon_32 Exon_33 Exon_34 Exon_35 Exon_36 Exon_37

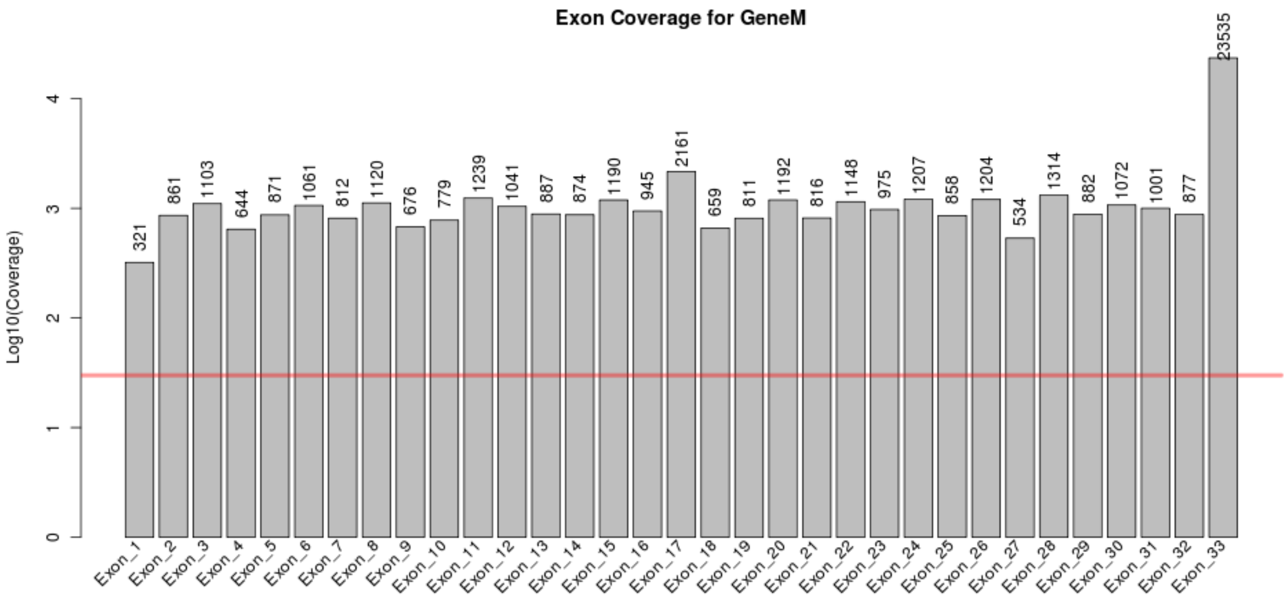
Exon Coverage for GeneM
Log10(Coverage)
0
1
2
3
4
321
861
1103
644
871
1061
812
1120
676
779
1239
1041
887
874
1190
945
2161
659
811
1192
816
1148
975
1207
858
1204
534
1314
882
1072
1001
877
23535
Exon_1
Exon_2
Exon_3
Exon_4
Exon_5
Exon_6
Exon_7
Exon_8
Exon_9
Exon_10
Exon_11
Exon_12
Exon_13
Exon_14
Exon_15
Exon_16
Exon_17
Exon_18
Exon_19
Exon_20
Exon_21
Exon_22
Exon_23
Exon_24
Exon_25
Exon_26
Exon_27
Exon_28
Exon_29
Exon_30
Exon_31
Exon_32
Exon_33

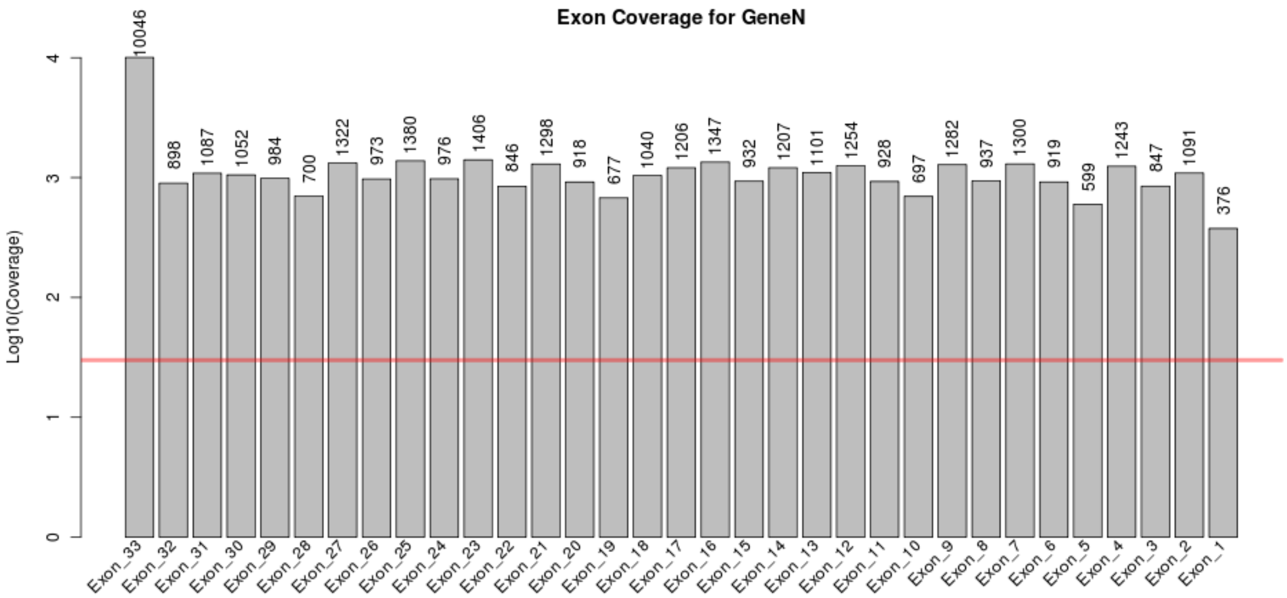
Exon Coverage for GeneN
Log10(Coverage)
0
1
2
3
4
10046
898
1087
1052
984
700
1322
973
1380
976
1406
846
1298
918
677
1040
1206
1347
932
1207
1101
1254
928
697
1282
937
1300
919
599
1243
847
1091
376
Exon_33
Exon_32
Exon_31
Exon_30
Exon_29
Exon_28
Exon_27
Exon_26
Exon_25
Exon_24
Exon_23
Exon_22
Exon_21
Exon_20
Exon_19
Exon_18
Exon_17
Exon_16
Exon_15
Exon_14
Exon_13
Exon_12
Exon_11
Exon_10
Exon_9
Exon_8
Exon_7
Exon_6
Exon_5
Exon_4
Exon_3
Exon_2
Exon_1

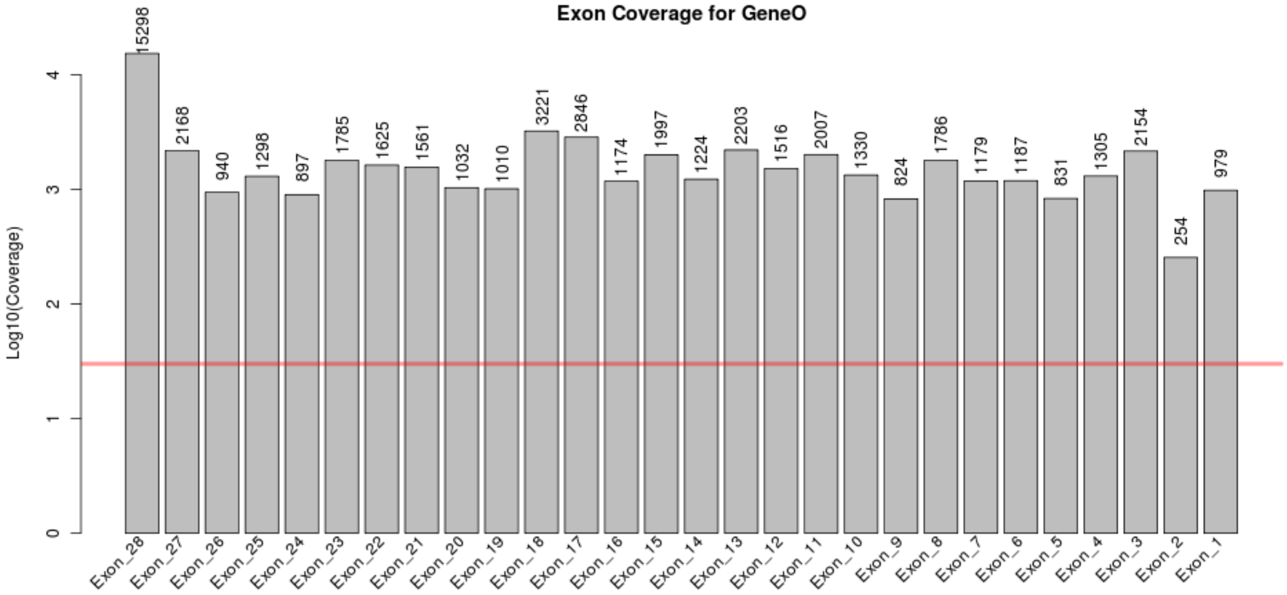
Exon Coverage for GeneO
Log10(Coverage)
0
1
2
3
4
15298
2168
940
1298
897
1785
1625
1561
1032
1010
3221
2846
1174
1997
1224
2203
1516
2007
1330
824
1786
1179
1187
831
1305
2154
254
979
Exon_28
Exon_27
Exon_26
Exon_25
Exon_24
Exon_23
Exon_22
Exon_21
Exon_20
Exon_19
Exon_18
Exon_17
Exon_16
Exon_15
Exon_14
Exon_13
Exon_12
Exon_11
Exon_10
Exon_9
Exon_8
Exon_7
Exon_6
Exon_5
Exon_4
Exon_3
Exon_2
Exon_1

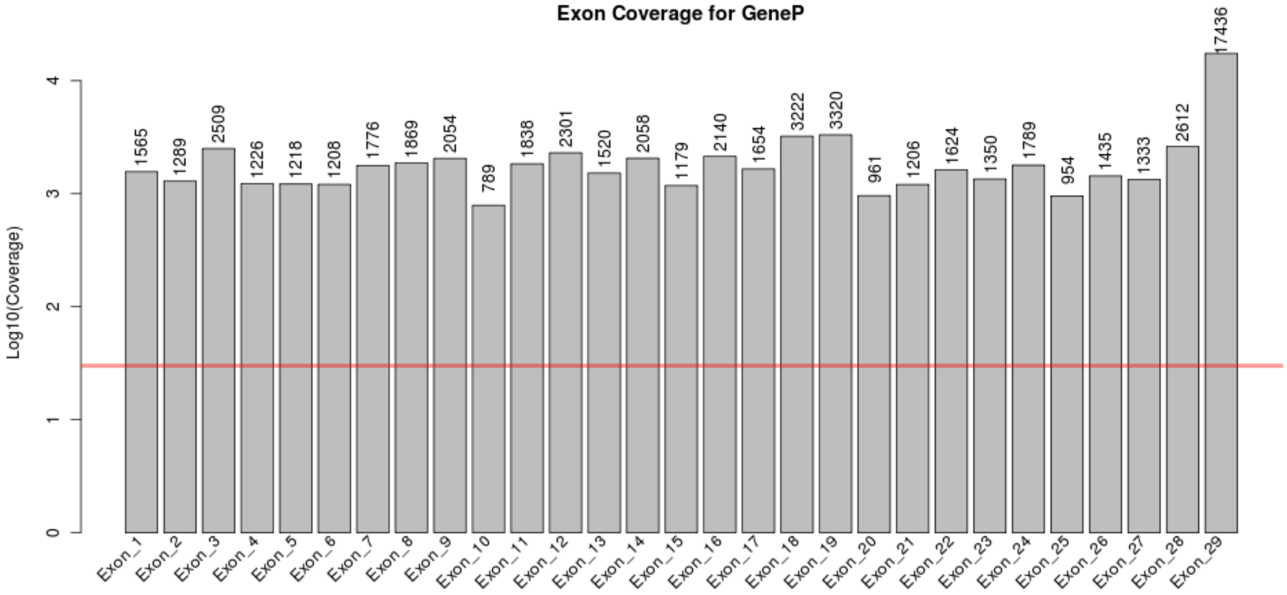
Exon Coverage for GeneP
Log10(Coverage)
0
1
2
3
4
1565
1289
2509
1226
1218
1208
1776
1869
2054
789
1838
2301
1520
2058
1179
2140
1654
3222
3320
961
1206
1624
1350
1789
954
1435
1333
2612
17436
Exon_1
Exon_2
Exon_3
Exon_4
Exon_5
Exon_6
Exon_7
Exon_8
Exon_9
Exon_10
Exon_11
Exon_12
Exon_13
Exon_14
Exon_15
Exon_16
Exon_17
Exon_18
Exon_19
Exon_20
Exon_21
Exon_22
Exon_23
Exon_24
Exon_25
Exon_26
Exon_27
Exon_28
Exon_29

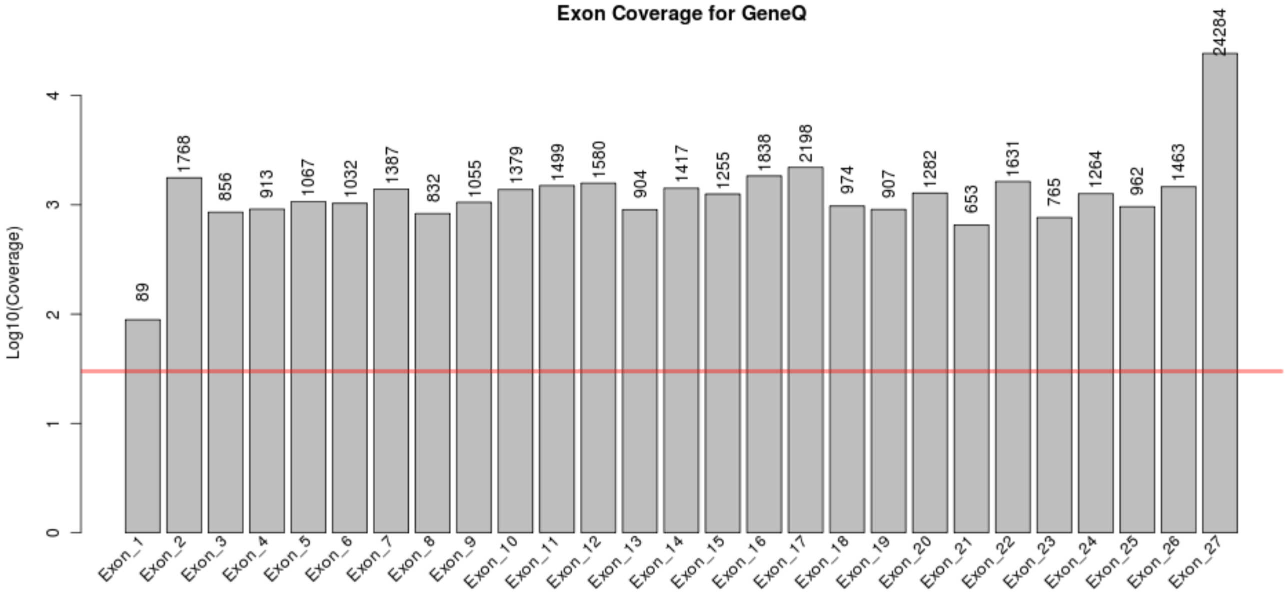

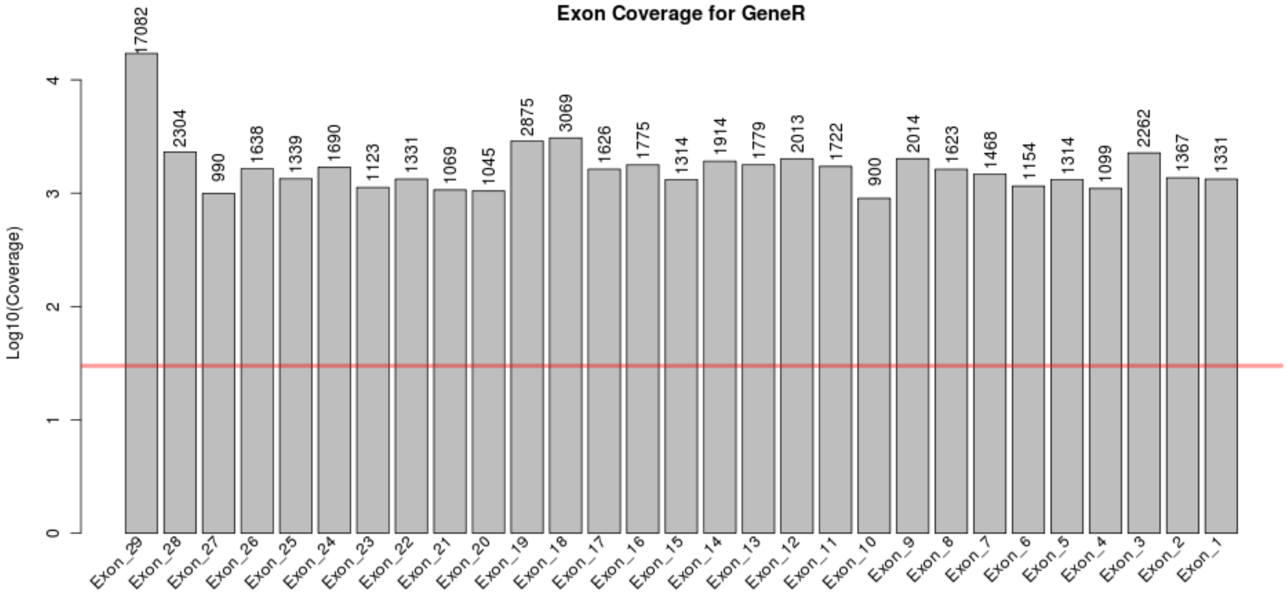

Failed Exon Plots

<u>NOTE:</u> ALL exons were tested for local coverage <30

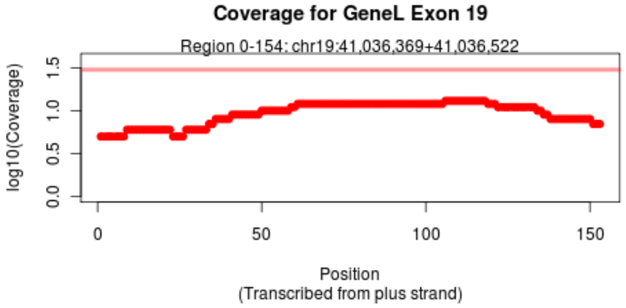

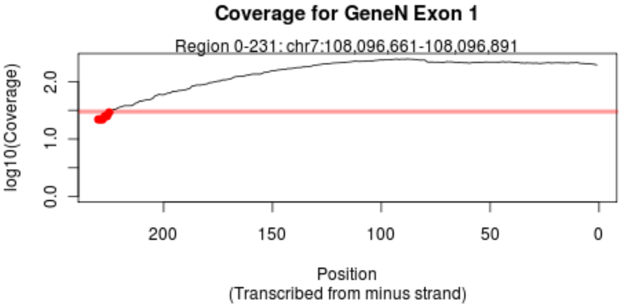

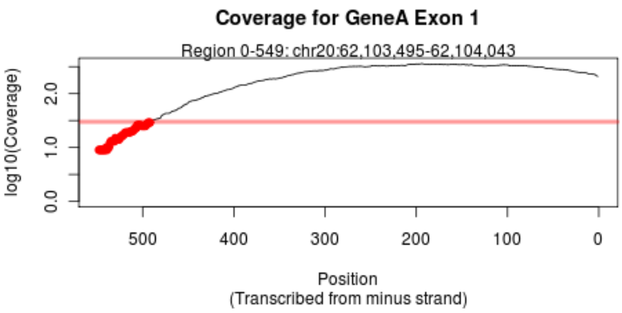
Coverage for GeneA Exon 1
Region 0-549: chr20:62,103,495-62,104,043
log10(Coverage)
0.0
1.0
2.0
500
400
300
200
100
0
Position
(Transcribed from minus strand)

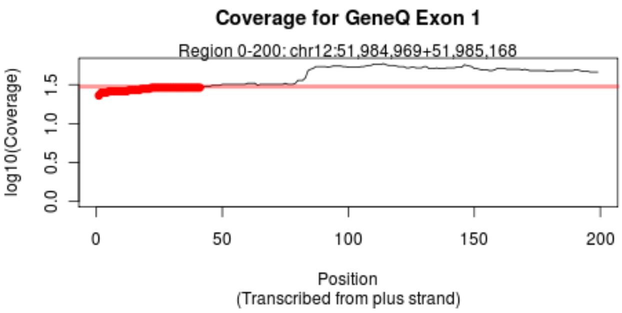
Coverage for GeneQ Exon 1
Region 0-200: chr12:51,984,969+51,985,168
log10(Coverage)
0.0
0.5
1.0
1.5
0
50
100
150
200
Position
(Transcribed from plus strand)

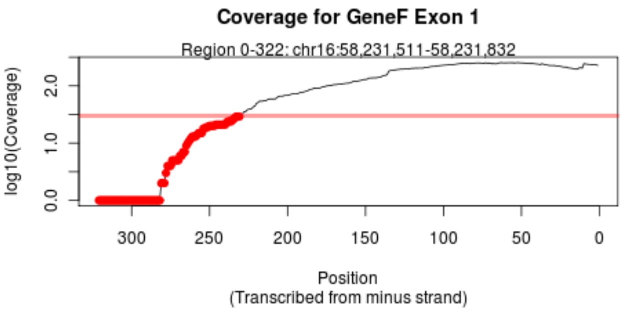
Coverage for GeneF Exon 1
Region 0-322: chr16:58,231,511-58,231,832
log10(Coverage)
0.0
1.0
2.0
300
250
200
150
100
50
0
Position
(Transcribed from minus strand)
