# Supplementary material for: A Robust Protocol to Increase NimbleGen SeqCap EZ Multiplexing Capacity to 96 Samples
Source: PLoS One. 2015 Apr 14;10(4):e0123872. doi: 10.1371/journal.pone.0123872 (PMC4397063; doi:10.1371/journal.pone.0123872)
Supplement: S2 Fig — For each gene a graph is depicted, showing the mean coverage per exon over all samples (n = 34). Error bars reflect ±1 standard deviation. The commonly used threshold of 30X coverage is indicated in each graph with a black line. (PDF) [file pone.0123872.s003.pdf]

S2 Figure. Coverage distribution per gene. For each gene a graph is depicted, showing the mean coverage per exon over all samples (n=34). Error bars reflect ±1 standard deviation. The commonly used threshold of 30X coverage is indicated in each graph with a black line.

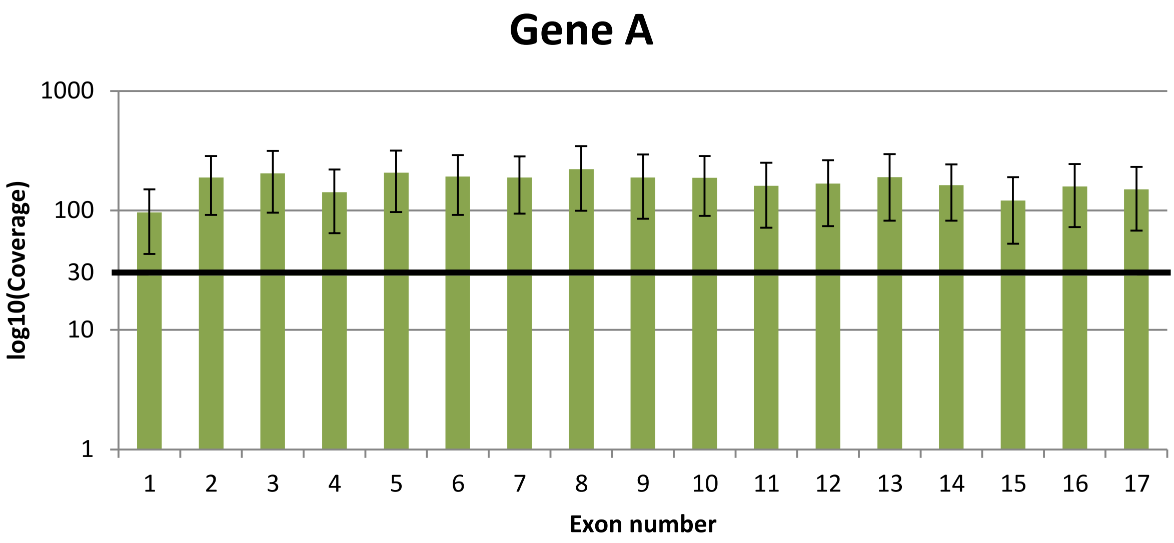

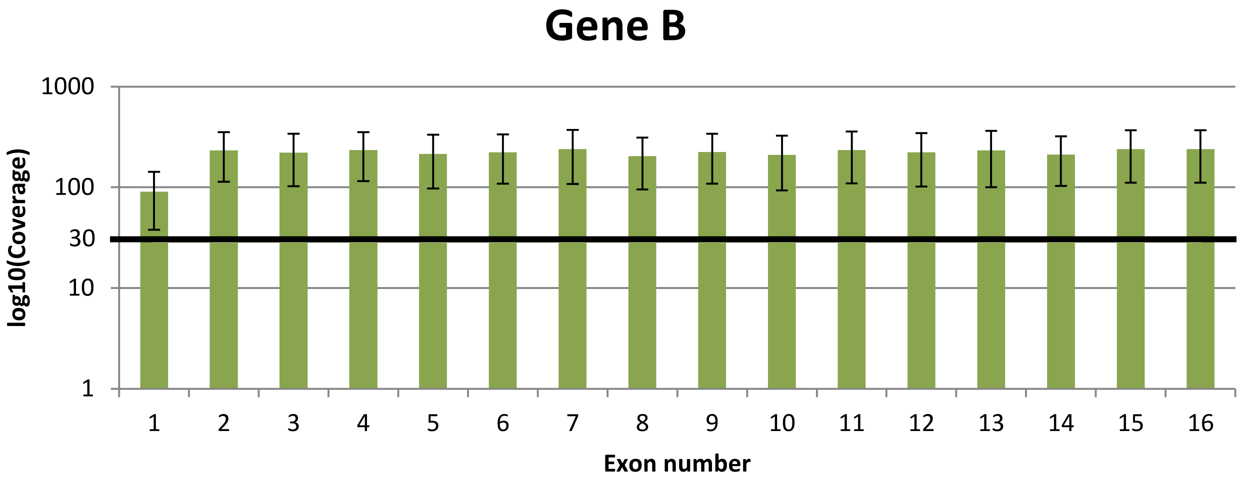

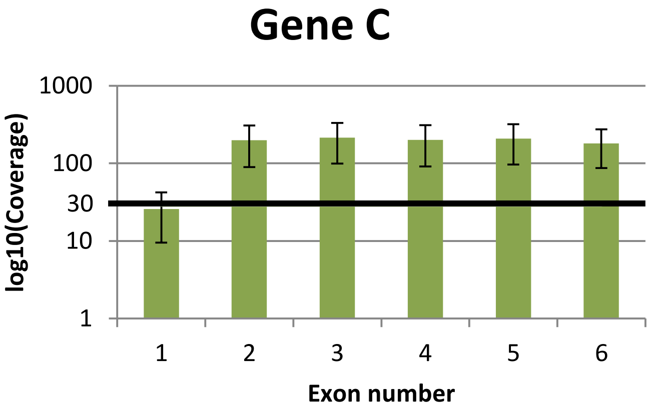

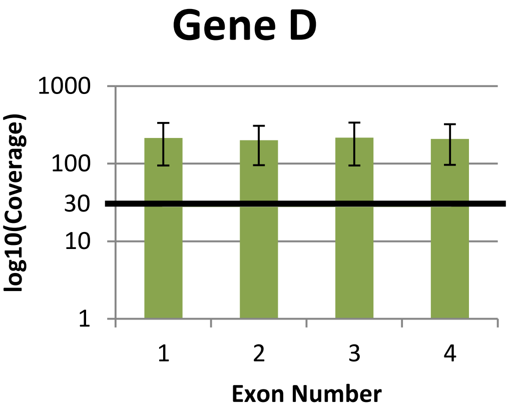

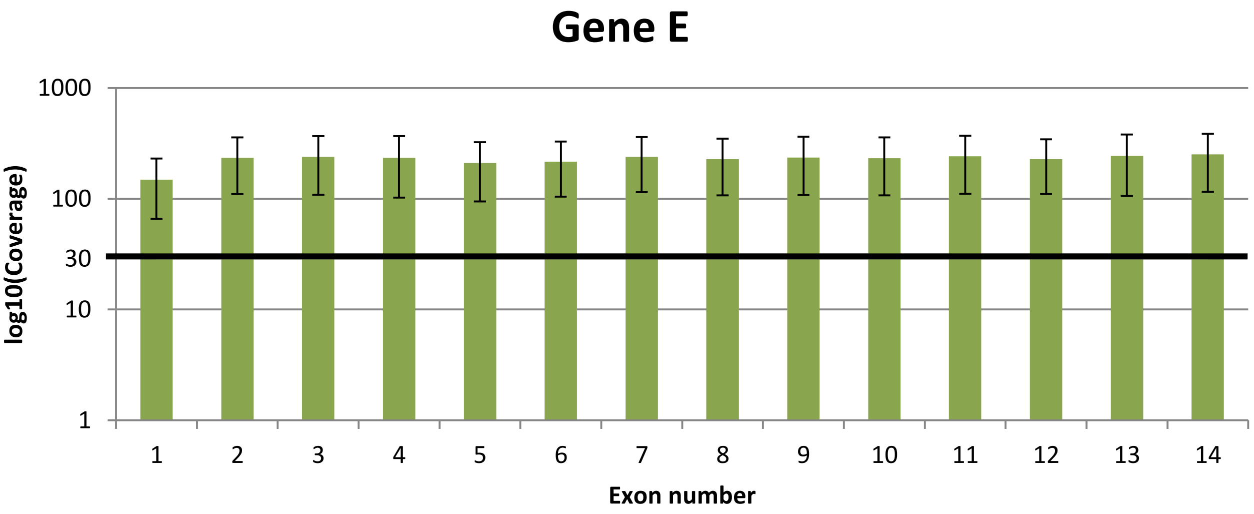
Gene E
log10(Coverage)
1000
100
30
10
1
1 2 3 4 5 6 7 8 9 10 11 12 13 14
Exon number

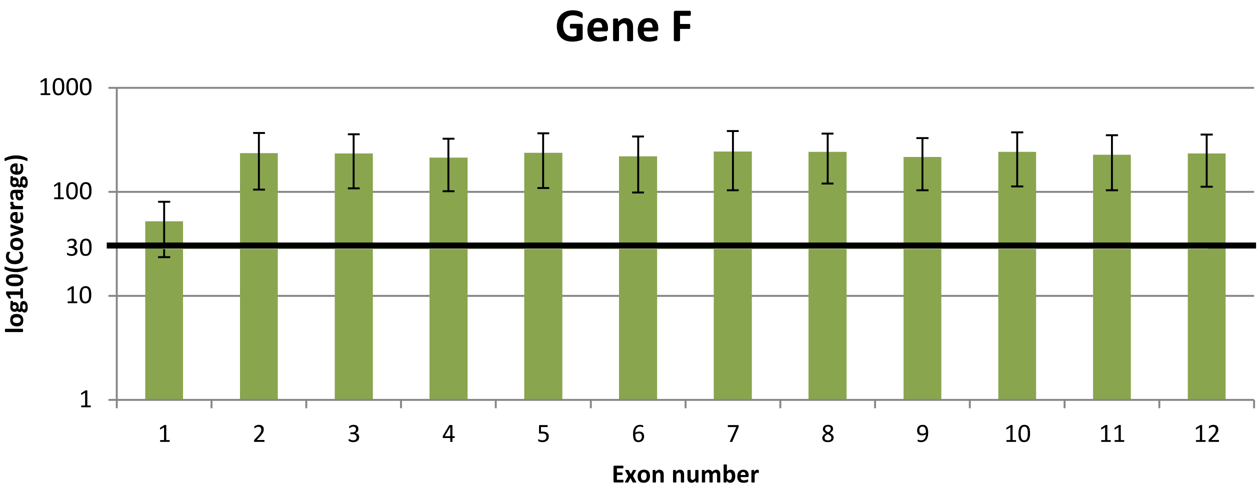
Gene F
log10(Coverage)
1000
100
30
10
1
1 2 3 4 5 6 7 8 9 10 11 12
Exon number

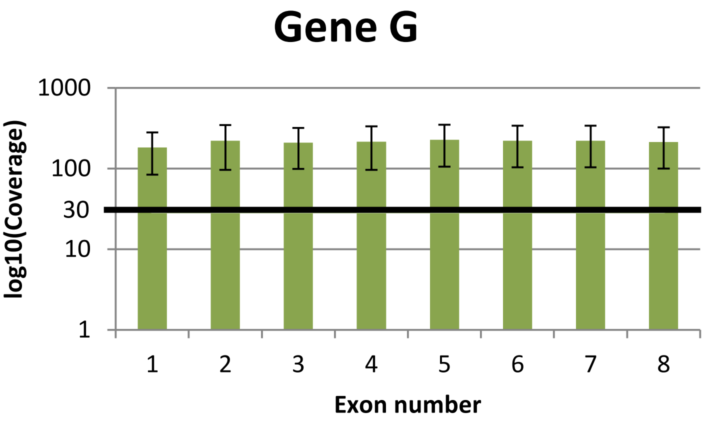
Gene G
log10(Coverage)
1000
100
30
10
1
1 2 3 4 5 6 7 8
Exon number

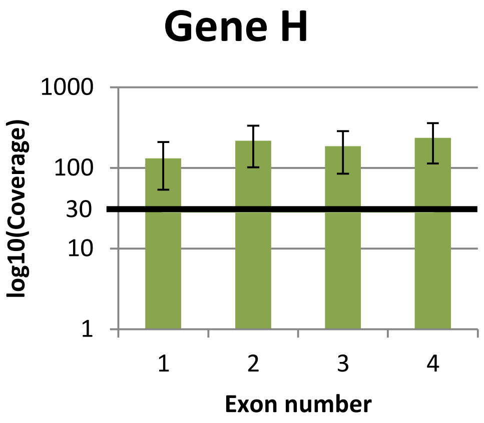
Gene H
log10(Coverage)
1000
100
30
10
1
1 2 3 4
Exon number

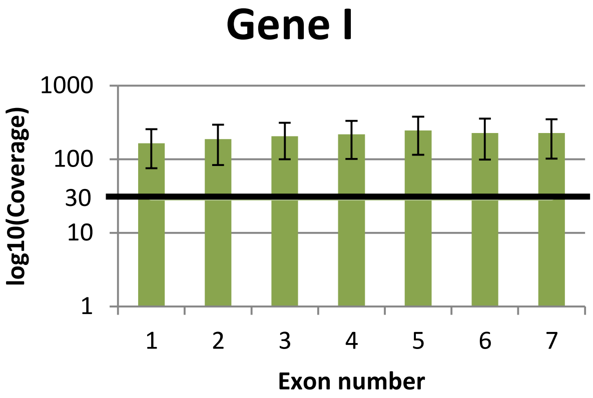
Gene I
log10(Coverage)
1000
100
30
10
1
1 2 3 4 5 6 7
Exon number

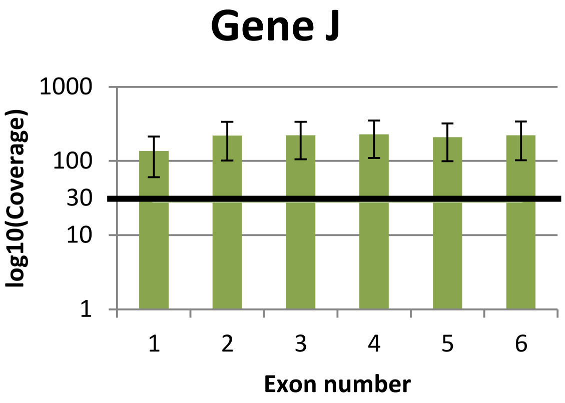
Gene J
log10(Coverage)
1000
100
30
10
1
1 2 3 4 5 6
Exon number

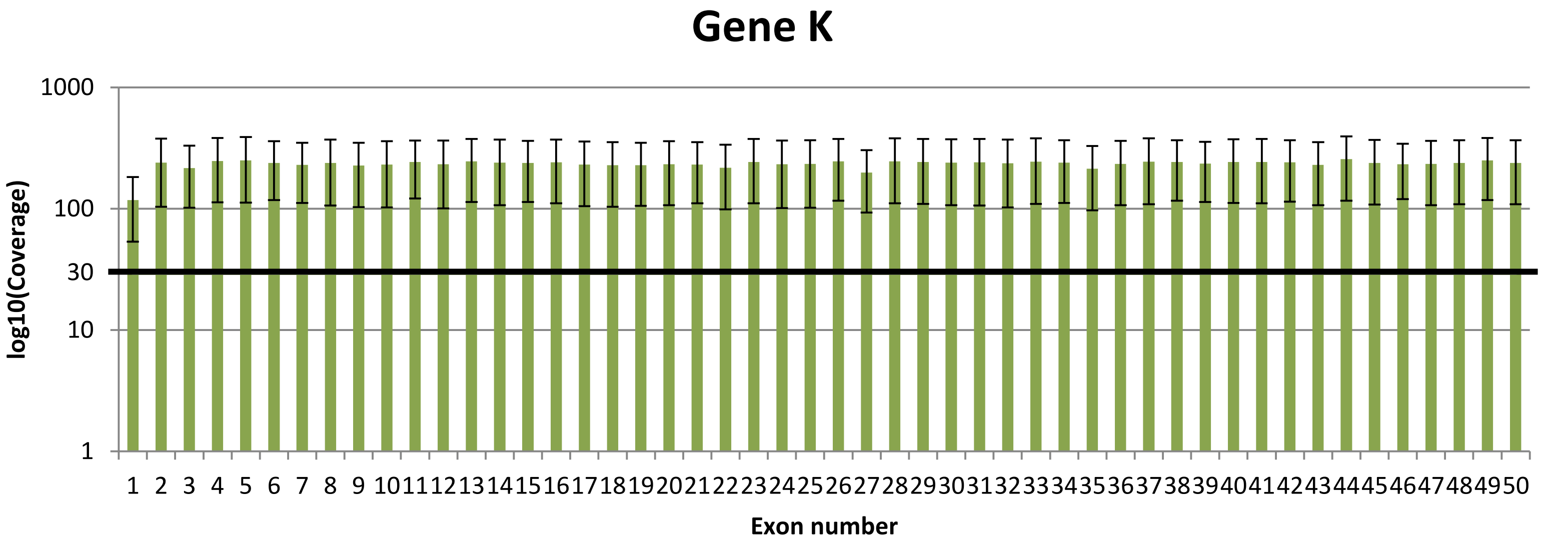
Gene K
1000
100
30
10
1
log10(Coverage)
1 2 3 4 5 6 7 8 9 10 11 12 13 14 15 16 17 18 19 20 21 22 23 24 25 26 27 28 29 30 31 32 33 34 35 36 37 38 39 40 41 42 43 44 45 46 47 48 49 50
Exon number

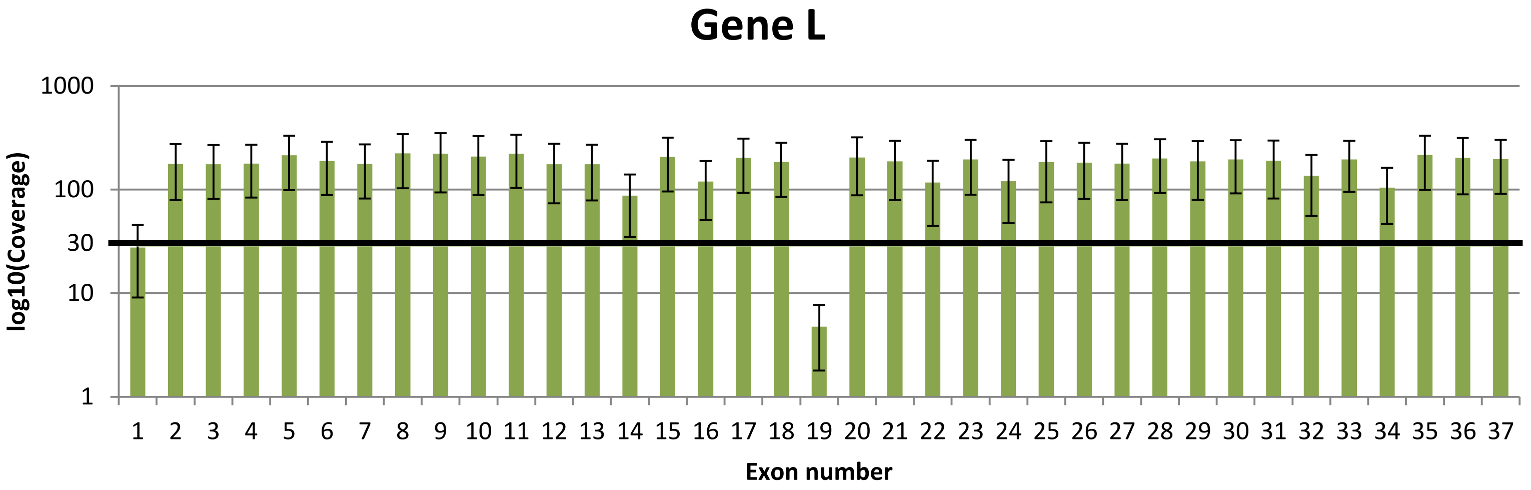
Gene L
1000
100
30
10
1
log10(Coverage)
1 2 3 4 5 6 7 8 9 10 11 12 13 14 15 16 17 18 19 20 21 22 23 24 25 26 27 28 29 30 31 32 33 34 35 36 37
Exon number

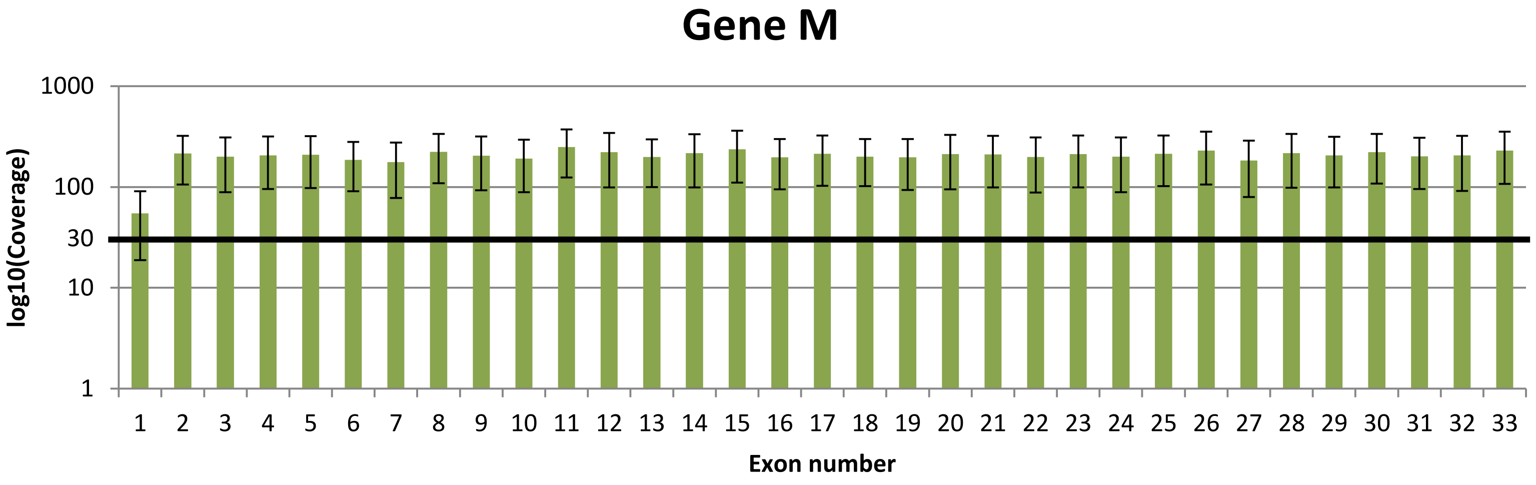
Gene M
1000
100
30
10
1
log10(Coverage)
1 2 3 4 5 6 7 8 9 10 11 12 13 14 15 16 17 18 19 20 21 22 23 24 25 26 27 28 29 30 31 32 33
Exon number

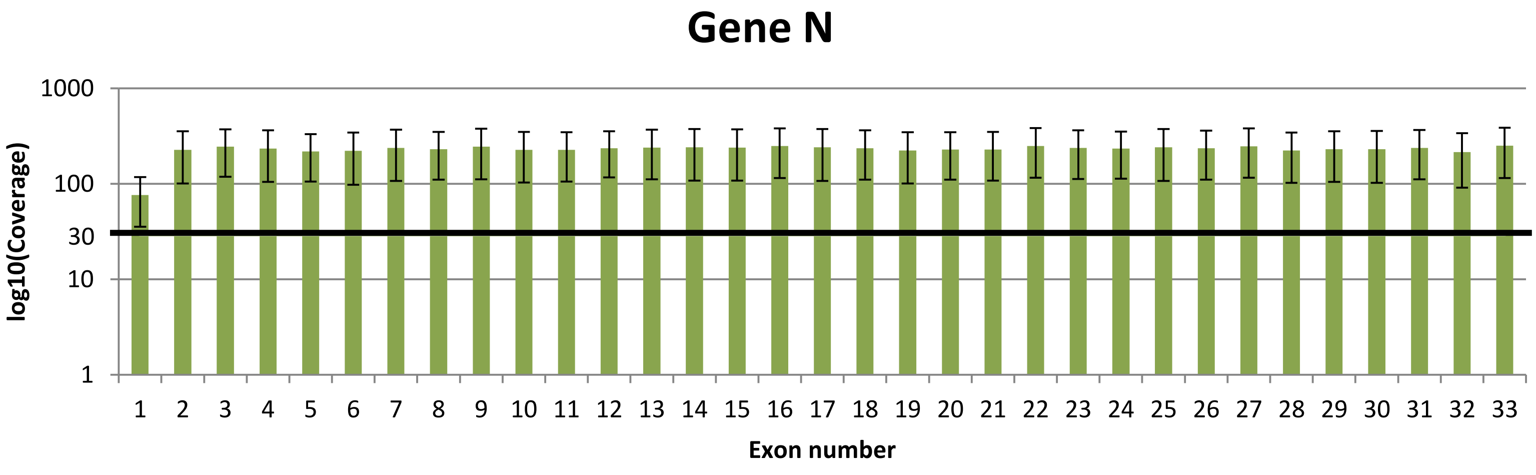
Gene N
1000
100
30
10
1
log10(Coverage)
1 2 3 4 5 6 7 8 9 10 11 12 13 14 15 16 17 18 19 20 21 22 23 24 25 26 27 28 29 30 31 32 33
Exon number

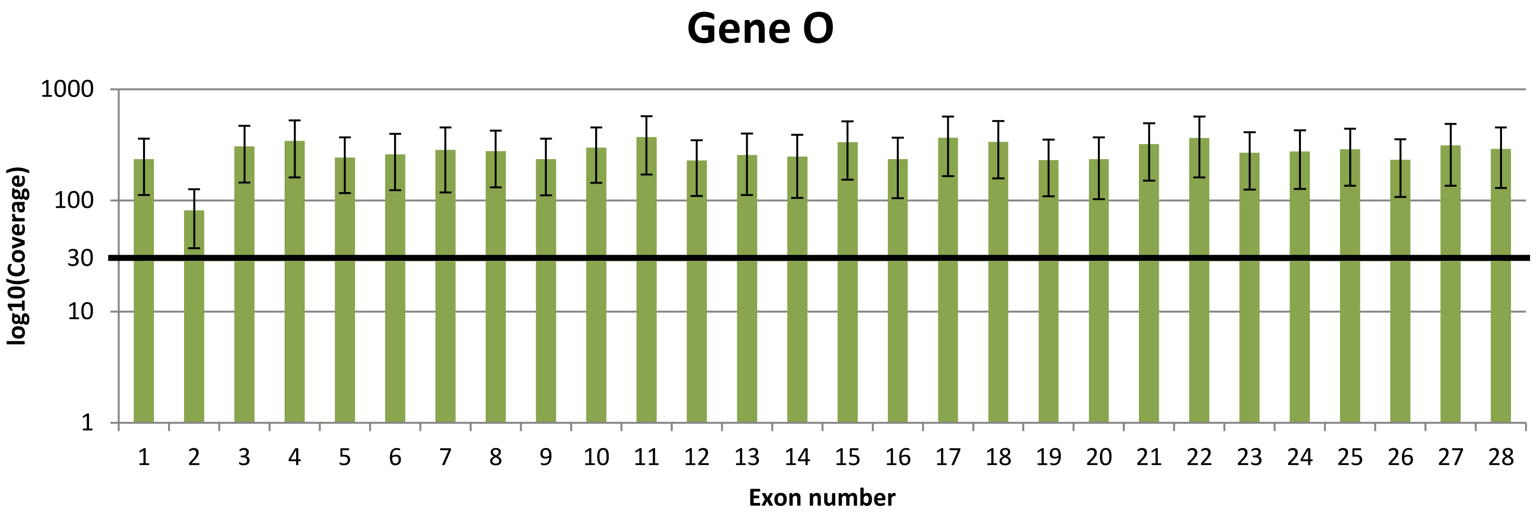
Gene O
log10(Coverage)
1000
100
30
10
1
1 2 3 4 5 6 7 8 9 10 11 12 13 14 15 16 17 18 19 20 21 22 23 24 25 26 27 28
Exon number

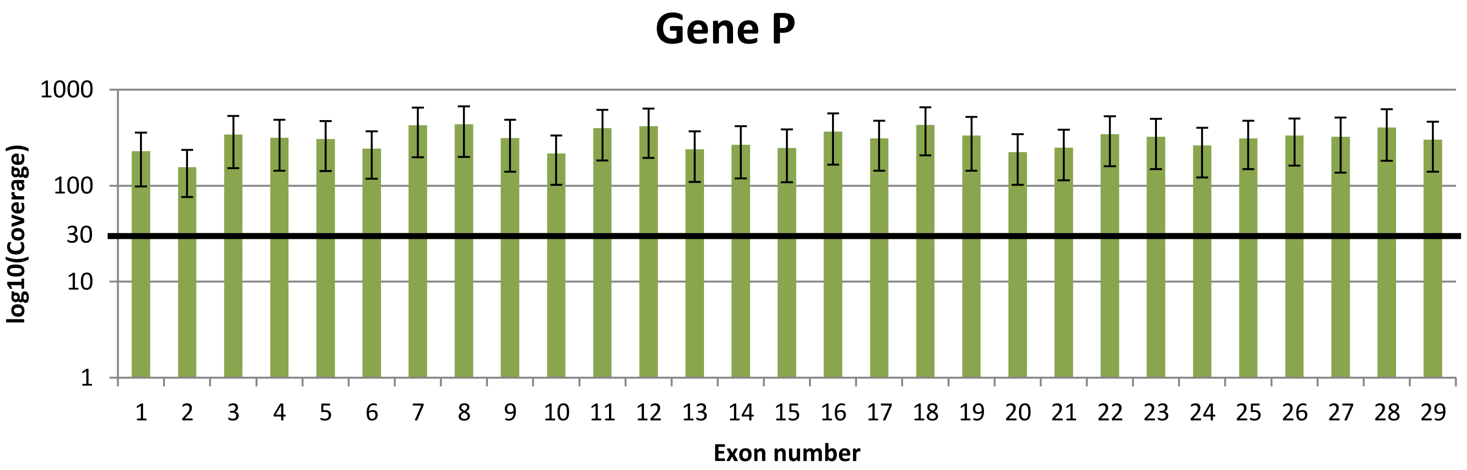
Gene P
log10(Coverage)
1000
100
30
10
1
1 2 3 4 5 6 7 8 9 10 11 12 13 14 15 16 17 18 19 20 21 22 23 24 25 26 27 28 29
Exon number

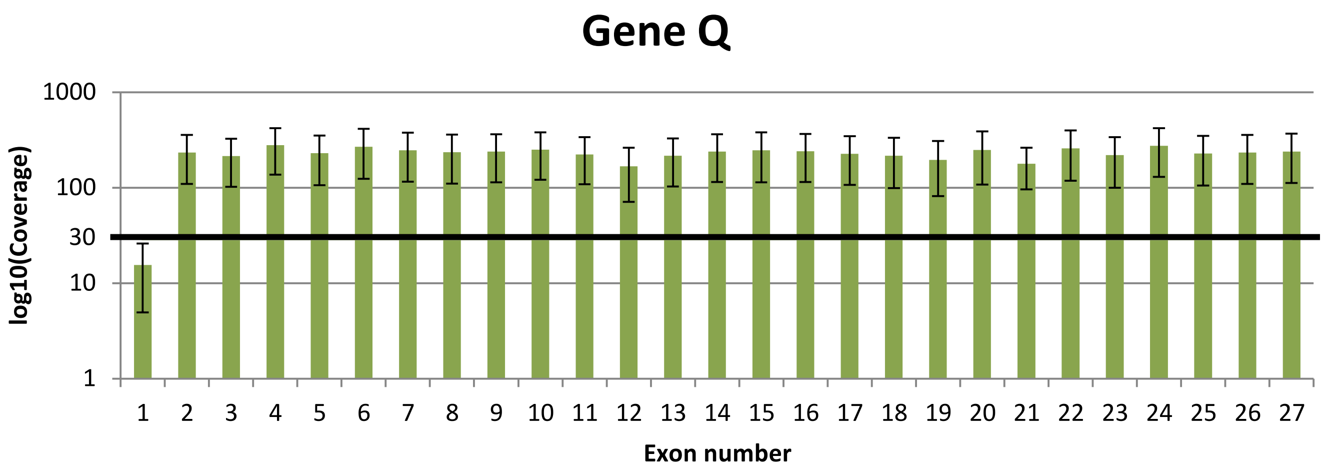
Gene Q
log10(Coverage)
1000
100
30
10
1
1 2 3 4 5 6 7 8 9 10 11 12 13 14 15 16 17 18 19 20 21 22 23 24 25 26 27
Exon number

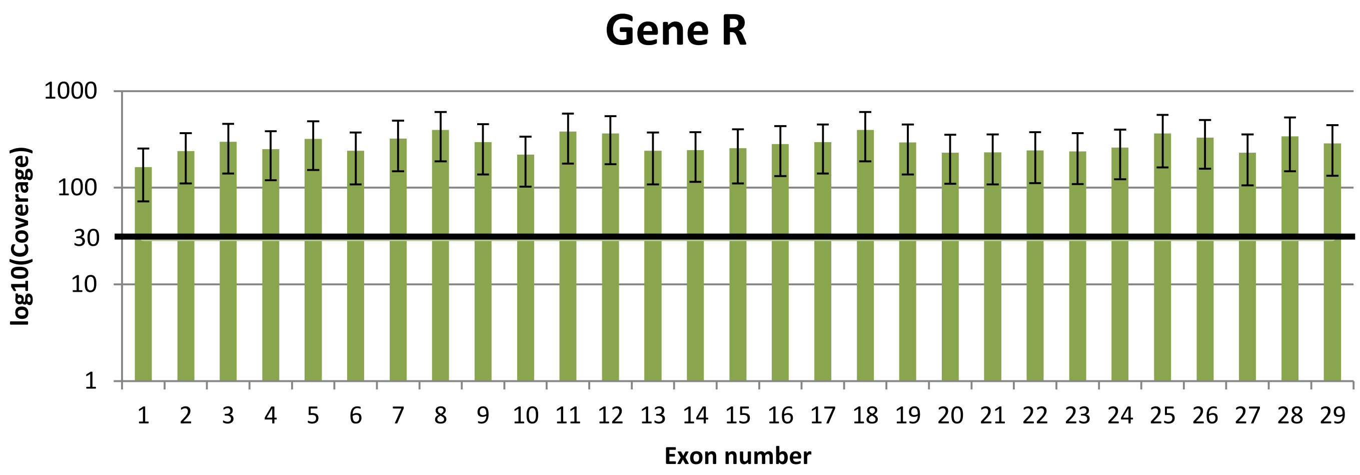
Gene R
log10(Coverage)
1000
100
30
10
1
1 2 3 4 5 6 7 8 9 10 11 12 13 14 15 16 17 18 19 20 21 22 23 24 25 26 27 28 29
Exon number
