# Supplementary material for: A Robust Protocol to Increase NimbleGen SeqCap EZ Multiplexing Capacity to 96 Samples
Source: PLoS One. 2015 Apr 14;10(4):e0123872. doi: 10.1371/journal.pone.0123872 (PMC4397063; doi:10.1371/journal.pone.0123872)

**S3 Figure. Cost-comparison of Nimblegen single-indexing vs. the presented dual-indexing protocol**

Costs include KAPA library preparation kits, adapter and blocking oligo costs, all SeqCap EZ products required for enrichment and the MiSeq v2 300 cycles sequencing kits (all prices based on the catalogue prices for 2014 in Belgium).

Standard protocol includes:
- Pre-enrichment pooling of 4 samples
- Single-indexing
- A total of 24 samples in one MiSeq v2 2x150 bp sequencing run

Adjusted protocol includes:
- Pre-enrichment pooling of 12 samples
- Dual-indexing
- A total of 96 samples in one MiSeq v2 2x150 bp sequencing run

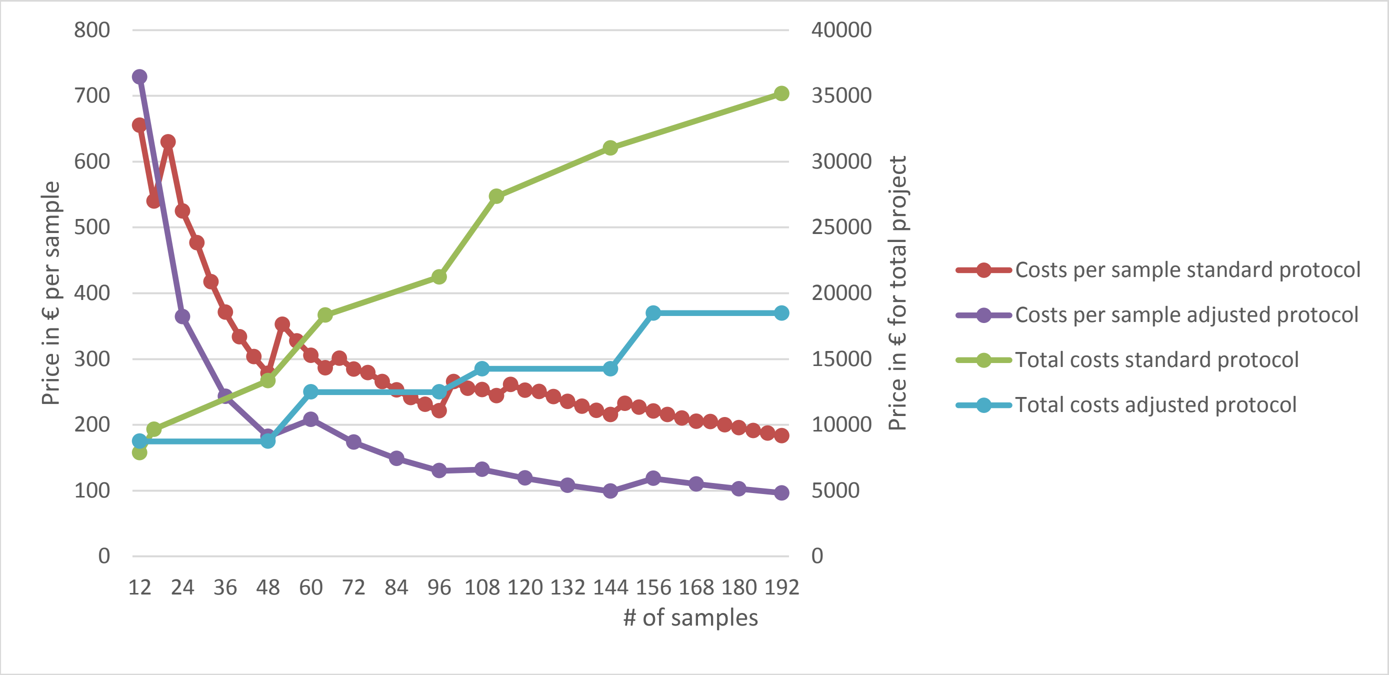

Supplement: S3 Fig — (PDF) [file pone.0123872.s004.pdf]
